# X-ray, Cryo-EM, and computationally predicted protein structures used in integrative modeling of HIV Env glycoprotein gp120 in complex with CD4 and 17b

**DOI:** 10.1016/j.dib.2016.01.001

**Published:** 2016-01-12

**Authors:** Muhibur Rasheed, Radhakrishna Bettadapura, Chandrajit Bajaj

**Affiliations:** Computational Visualization Center, Institute of Computational Engineering and Sciences, The University of Texas at Austin, Austin, TX, USA

## Abstract

We present the data used for an integrative approach to computational modeling of proteins with large variable domains, specifically applied in this context to model HIV Env glycoprotein gp120 in its CD4 and 17b bound state. The initial data involved X-ray structure PDBID:1GC1 and electron microscopy image EMD:5020. Other existing X-ray structures were used as controls to validate and hierarchically refine partial and complete computational models. A summary of the experiment protocol and data was published (Rasheed et al., 2015) [26], along with detailed analysis of the final model (PDBID:3J70) and its implications.

**Specifications Table**

**Table:** 

Subject area	*Chemistry, Biology, Computer Science*
More specific subject area	*Structural molecular biology, Protein modeling, Molecular structure prediction*
Type of data	*Protein structures (PDB format),Tables, charts, figures* etc.
How data was acquired	*Input for the integrative modeling pipeline was acquired from public databases- the protein data bank, and the electron microscopy data bank. Preliminary stage models were produced using Swiss-Model and I-TASSER software. Structural quality of models were evaluated using a number of software including MolProbity, ERRAT, PROSA II, ModEval, PDB validation suite, Verify3D, ProCheck and PSVS, a suite which computes a set of quality scores including some of the ones mentioned here. Energy minimization and minor structural refinement was done using the KoBaMin server. Conformational search, assembly, and assessment of quaternary contact quality were performed using F*^*2*^*Dock and MolEnergy software suites. Correlation of atomic model and electron microscopy data was carried out using the PF*^*2*^*Fit software. We used R for statistical analysis, and TexMol and PyMol for visualization. Detailed citations for these software are included in the article.*
Data format	*Raw: Computationally predicted models (PDB format) Analyzed: Molecular properties of models; charts, figures etc.*
Experimental factors	*The protocol used to resolve the complete structure of gp120 in complex with CD4 and 17b involved multiple steps, each involving computational clustering and pruning of data. Please see the main article*[26]*for details, as well as the brief description in the body of this article. Note that, this is a computational modeling protocol and there was no experimental pretreatment of samples in the traditional sense.*
Experimental features	*We pose the problem of computational modeling of a protein as- given the primary sequence of a protein, a set of available partial structures at atomic resolution and additional data including possible binding sites, electron microscopy (EM) maps etc, report atomic structure of the entire protein such that it explains (fits) the given data and maximizes a scoring function. The scoring function is designed to reflect structural quality at secondary, tertiary and quaternary levels.*
Data source location	*Not applicable*
Data accessibility	*All data is publicly accessible with no restrictions. All data necessary to understand and replicate the entire pipeline, or any part of it, is provided as a compressed folder with this paper.*

## Value of the data

• The data is sufficient to reproduce the computational model of the structure of gp120 bound with CD4 and 17b contains refined models of the V1-V2 and V3 loops [26]**.**
• Furthermore, the data, including partial and unrefined models from initial stages of pipeline, would enable researchers to quickly examine alternate protocols and refinement techniques to possibly achieve better models.
• The initial stage model data can also be a valuable starting point for modeling gp120 and its variable loops in complex with other partners.

## 1. Data

The data accompanying this paper is organized according to the modeling pipeline stages. The stages are- (1) setting up controls and calibration of software (scoring model) using existing X-ray data; (2) generating an ensemble of complete models for gp120 by homology modeling; (3) clustering and selecting small set of candidate models for each fragment; (4) fragment assembly and refinement in complex; and (5) analysis and validation of refined model. The dataset includes relevant information, raw data and analysis for each of these stages.

1. The *existingXrayModels* folder contains a summary of the structural properties of all existing (deposited into protein data bank prior to our modeling exercise) X-ray structures, statistical analysis of their similarity, a description of the missing residues in each model, and a structure based clustering which shows that structural similarity is dictated more by similarity of binding partners, than similarity of the sequence. Note that the raw PDB data is not provided since they are already available from the protein data bank using the accession codes we provide.
2. The folders *swissModels* and itasserModels contain models (in PDB format) generated by the Swiss-Model and ITASSER software using a slew of different partial gp120 X-ray structures as templates. They produced a wide range of conformations, especially for the variable regions V1, V2 and V3 of gp120. However, the models did not have sufficiently high quality, both in terms of tertiary structure and quaternary interactions with binding partners CD4 and 17b.
3. The folder *fragments* contains structural models (in PDB format) of a small set of fragments extracted from the swiss and itasser models. The set was selected by clustering and then picking high scoring models from each cluster, unless a cluster only had poor scoring models.
4. The *splicedModels* folder contains all models generated by assembling different fragments. The *optimizedModels* contains a subset of the spliced models that were selected by clustering and then picking high scoring models from the clusters. These were energy minimized to produce the models in this folder. model 31 had the best scores and also had better binding specificity and was chosen as the final model. Each of these models are provided in PDB format.
5. Details of the final model of the gp120+gp41+CD4+17b complex is placed in *finalModelAndValidationData* folder. The structure itself can be accessed in RCSB PDB with PDB id: 3J70.
6. Computationally determined quality metrics, clustering, and filtering details related to different stages of the protocol are given in the *scoringData* folder.

## 2. Experimental design, materials and methods

Here we discuss different aspects of the modeling protocol and its application in modeling gp120. Under each section we also refer to the corresponding dataset that accompany this paper.

### 2.1 Model evaluation criteria

Our computational protocol relies upon a scoring model, which was calibrated using all existing X-ray structures of gp120 to distinguish correctly and incorrectly folded structures as well as correct and incorrect binding configurations.

The secondary and tertiary structural quality of a model is evaluated using a set of currently available tools with complementary properties. The tools include Verify3D [19], PROCHECK [21], ERRAT [8], ProSA [31], and MolProbity [9]. Among the tools, PROCECK and MolProbity focuses on local stereochemistry, and Verify3D, ERRAT, ModEval etc. focus on tertiary folds and global 3D quality metrics. Hence, a model that scores high in all of these metrics is quite reliable. We used the PDB evaluation suite ADIT, Modeval [30] and Qmean z-score [2] for independent validation of the final model, but not during the scoring and search. A weighted combination of confidence scores (z-scores) for these terms is defined as *s*_*internal.*_

To measure the quaternary structure quality, for proteins in a complex, we use the scores *s*_*external*_ consisting five terms. The clash and severe clash scores are the number of atoms of one protein whose center lies, respectively, too close and inside the VDW volume of any atom of the other. We expect zero severe clashes, and fewer than 10 clashes in a good model. The interface area term is defined as the area of the molecular surface of one protein that is within 2 Å from any point on the molecular surface of another. There is no global rule of thumb for interface area, and one must calibrate the range of acceptable values or an expected value based on some known interfaces (the calibration is described in the next subsection). We also define a score that is computed as a sum over the contact potentials for each residue-residue pairs that are in contact. We use the contact potentials reported by Glaser et al. [11], where positive and negative potentials indicate, respectively, higher and lower probability of the finding such contact on an interface. All of the scores mentioned here were previously calibrated for protein-protein docking predictions [7] and were found to be quite discriminatory, especially for antibody-antigen complexes. Finally, when a corresponding cryo-electron microscopy map is available, we use the external-total ratio (ETR) [25] and he mutual information score (MIS) [29], [33] to evaluate the quality of fit of the model with the EM map. The first is minimized when the model is completely inside the EM map, the latter is maximized when the model has larger overlap with the map.

### 2.2 Calibration of scoring functions

We collected structures of gp120 currently deposited in the PDB (the data can be found under the existing XrayModels folder) and computed raw scores for the scoring terms for each of them. Then the mean (*μ*), min (*m*), max (*M*) and standard deviation *σ* of these raw scores were used to define z-scores. Note that, for the terms in *s*_*internal*_, a similar calibration was reported by Bhattacharya et al. [4] over large a benchmark of crystal and NMR structures with less than 50% similarity with each other. gp120 is among the largest of the proteins considered in the dataset of Bhattacharya et al. and the overall scores the existing PDB models of gp120 got is lower than the expected, and in general corresponded to lower resolutions. We found that *s*_*internal*_ can correctly distinguish between low and high resolution crystal structures within the control set. The Pearson correlation coefficient of *s*_*internal*_ and corresponding resolutions across the 20 models in the control set is −0.5927 (a tail probability of 0.006175). The correlation is statistically significant and hence if the *s*_*internal*_ of a model is higher than the average value (–8.14) of the control set, we can accept it, with high confidence, as a high resolution and stereochemically accurate model. See detailed data in internalscores.xls.

For the external terms, we tweaked the definition of z-score a little. Z-score is normally defined as deviation from mean. However, since the complete gp120 models we want to predict have more than 100 extra residues as compared to the partial models in the control set, some of the raw scores are expected to be skewed, and not be close to the mean of the control set. For instance, the interface area, MIS (which is maximized when larger portion of the density map is covered by a model upon fitting) and residue-contacts (negative and positive) for the complete models are expected to be higher than all the models in the control set. So, we use the extreme values of the control set as the expected value while computing z-scores for these terms. Note that as far as ranking and comparison of models are concerned this is no different from using the mean. We found that *s*_*external*_ correctly distinguishes between gp120 models that were co-crystallized with CD4 and 17b and hence have a correct site topology, from ones that were not. See detailed data in externalscores.xls.

### 2.3 Summary of available models

Currently available X-ray structures of gp120 in complex with CD4 and 17b are missing all variable domains of gp120 which detracts from getting a complete understanding of their effect on the binding interactions. We found that a few researchers had previously tried to model the variable regions using computational tools without success. The publicly available models from such attempts are provided with this paper in the folder existingHomologyModels. None of these models are well-folded.

### 2.4 Initial stage modeling

We started our pipeline by using Swiss-model [28] and I-TASSER [27], two state of the art protein modeling software that have additionally performed well at CASP challenges. We used UniProtKB sequence P04578 (the same sequence as 1GC1:Chain G) to produce models which contained the variable regions, the sequence alignment and template identification tools built into these two modelers picked 3JWD and 4NCO as templates. However, neither the model produced by Swiss-model nor the 5 models produced by I-TASSER scored within 2 standard deviations of the expected *s*_*external*_ and *s*_*internal*_ values. To get a better coverage of the possible models, we generated more models (61 in total) by manually selecting different templates (gp120 cores from different crystal structures). All these models are provided in the swissModels and itasserModels folders. Their scores can be found in the internalscores.xls and externalscores.xls files.

We found that in most of these models, the primary reason of the low scores was that the variable loops were folded in such a way that the overall structure of gp120 was compact and low energy. However, this occluded the binding sites of CD4 and 17b, hence scores poorly in terms of quaternary terms. This highlights the fact that unless the EM density map and/or the neighboring proteins are considered at while modeling a protein, the resulting low energy state may not be the native one. Several figures included in the data show the structures, their diversity, and their inclination to occlude the binding sites.

### 2.5 Fragment based modeling

We decomposed each of the initial models into fragments (core, V1V2, V3, V4, C-termini and N-termini), and clustered them based on similarity under TM-score [34]. The fragments were scored individually and high scoring members from each cluster were selected. We found that even some initial models with poor scores, had a fragment that scored high, when considered in isolation. The fragments can be found in the fragments folder in the dataset accompanying this paper.

A set of models for gp120 were prepared by assembling the fragments in all possible combinations. While these assembled structures were not stereochemically sound as the bond lengths/angles at the joint are too far from ideal at the beginning, after local structural refinement and energy minimization, the stereochemical and energetic quality of the models are significantly improved. Also, *s*_*external*_ scores also much better than the ones generated in the initial stage. The assembled models are provided in the splicedModels folder.

### 2.5 Optimized models

We clustered the spliced models based on TM-scores and picked highest croing ones from each cluster. We first applied our docking [7] and fitting [3], [1] protocols to improve the relative configuration of gp120, CD4 and 17b with each other as well as with respect to the EM map EMD5020. As a result, the score for almost all the terms improved significantly. Also all the optimized models have very good energetics and *s*_*internal*_ scores. However, two models (Model20 and Model23) have quite poor *s*_*external*_ scores. Model31 and Model35 both are assessed as high quality in terms of both *s*_*internal*_ and *s*_*external*_ scores, and considered medium quality in terms of energy. Note that high quality means that they scored better than average crystal structures in our control, and according to the calibration and correlation mentioned before, their qualities are equivalent to resolutions better than 3.5 Å. Detailed data can be found in the interscores.xls and externalscores.xls files and in the plots under the plots folder. The models can be found in the optimizedModels folder.

### 2.6 Binding site analysis

The binding site analysis was performed by docking CD4 and 17b with the optimized models using F2Dock [7]. F2Dock reports the top 1000 possible binding poses. We define the parts of the surface of gp120 that is in contact with the CDR loops of CD4 and 17b in a docking pose as the footprint/site of that particular pose. For each point on the surface of gp120 model, we compute the ratio of the number of poses whose footprint includes the point, and the total number of poses as the probability of that point being on the binding site. The binding site score is then defined as the sum of the probabilities of all the points on the surface that would be in contact with CD4 (or 17b) in their native poses. In other words, a model that has high likelihood of having the binding site at the correct region, scores high. Model31 had more specificity near the correct CD4 and 17b binding sites compared to model35. The data can be found in the bindingSiteData folder.

### 2.7 Analysis of the final model

Ramachandran plot analysis by Procheck [21] showed 89.6% residues in most favored, 7.6% in additionally allowed, 1.4% in generously allowed and only 1.4% in disallowed regions. Overall Procheck g-factor is −0.22 for *φ –ψ* angles only and −0.04 for all, both of which is extremely favorable and correspond to high resolution (<2 Å) structures [4]. In total 10 band-contacts were reported and 3.6% residues were found to have bad planarity. ProsaII composite score [31] for the model is 0.76, which is also representative of high resolution structures [4]. MolProbity [9] composite score for the model is 30.44 (z-score −3.70), which in general indicates that a model is in the low resolution range. However, we note that among existing X-ray models of gp120, 1G9M, 1G9N, 1GC1, 1RZJ and 3RJQ all have worse MolProbity scores. Verify3D [19] reports that more than 71% of the residues have a 1D–3D score above 0.2, which is in acceptable range according to Verify3D׳s guidelines.

We used the PDB validation software (ADIT), Modeval [30] and Qmean z-score [2] to provide independent validation of the quality. PDB validation software (ADIT) reports RMS deviation for bond angles at 0.7 degrees and bond length deviation of 0.003 Å, both of which is quite acceptable. ModEval predicted an RMSD of 3.378 (for the gp120 chain only). The Qmean z-score was 1.666 which is within the acceptable range for a protein of this size. Detailed data and figures can be found in the folder finalModelAndValidationData.

### 2.8 Predictions derived from

We computed the footprint of different antibodies whose X-ray structures in bound state with gp120 (core or complete) are available. We transformed the bound gp120-antibody complex such that the gp120 chain aligns with our gp120 model. Then computed the part of the surface of our gp120 which comes in contact with the antibody and the parts that intersect/overlap. A detailed list of the number of contacts and clashes for different antibodies as well as figures displaying such information is available in the folder bindingfootprints.

Chemical cross-linking is often used to generate low resolution distance constraints between parts of a protein (or multiple proteins). We used Xwalk [15], a computational tool that mimics cross-linking experiments by calculating the distance between two residues along the surface of the proteins, to identify inter-domain(gp120-CD4 and gp120-17b) cross-links in our model. We considered only cross-links between ARG, ASP, GLU and LYS residues whose C-beta atoms were within 25 Å of each other. As expected, we observed a high number of cross-links between residues at the CD4bs with CD4, and 17bbs with the heavy chain of 17b. However, we also identified a large number of crosslinks between CD4 and the V1V2 region, and a few cross-links between the light chain of 17b with the V3 region. The cross-links between 17b-CD4, and 17b-V1V2 were very few. The lack of predicted cross-link constraints between 17b and the V1V2 region, and the presence of high number of predicted cross-link constraints between CD4 and V1V2, may be considered as another structural explanation for the conformational motion of the loop, especially the preference of the V1V2 to move away from the 17b binding site (or by extension, the CCR5 binding site). The data is available in the folder crosslinks.
