# Supplementary material for: X-ray, Cryo-EM, and computationally predicted protein structures used in integrative modeling of HIV Env glycoprotein gp120 in complex with CD4 and 17b
Source: Data Brief. 2016 Jan 12;6:833–9. doi: 10.1016/j.dib.2016.01.001 (PMC4749890; doi:10.1016/j.dib.2016.01.001)
Supplement: Supplementary file 1 — Supplementary material [file mmc1.rtf]

No conflict
